# Linking Survey and Twitter Data: Informed Consent, Disclosure, Security, and Archiving

**DOI:** 10.1177/1556264619853447

**Published:** 2019-06-21

**Authors:** Luke Sloan, Curtis Jessop, Tarek Al Baghal, Matthew Williams

**Affiliations:** 1 Cardiff University, Cardiff, UK; 2 NatCen Social Research, London, UK; 3 University of Essex, Colchester, UK

**Keywords:** surveys, Twitter, linked data, ethics, consent, disclosive, archiving, reuse

## Abstract

Linked survey and Twitter data present an unprecedented opportunity for social scientific analysis, but the ethical implications for such work are complex—requiring a deeper understanding of the nature and composition of Twitter data to fully appreciate the risks of disclosure and harm to participants. In this article, we draw on our experience of three recent linked data studies, briefly discussing the background research on data linkage and the complications around ensuring informed consent. Particular attention is paid to the vast array of data available from Twitter and in what manner it might be disclosive. In light of this, the issues of maintaining security, minimizing risk, archiving, and reuse are applied to linked Twitter and survey data. In conclusion, we reflect on how our ability to collect and work with Twitter data has outpaced our technical understandings of how the data are constituted and observe that understanding one’s data is an essential prerequisite for ensuring best ethical practice.

## Introduction

The advent of social media has provided researchers with a potentially rich source of information regarding the behaviors, attitudes, and beliefs of individuals (Sloan, Morgan, Burnap, & Williams, 2015), but with it has come the substantial but necessary task of reconceptualizing some of the standard practices of ethical social research—voluntary participation (informed consent), minimizing harm (disclosure control and security), and maximizing value (archiving). The mass collection of data from social media platforms has enabled us to access hitherto unprecedented volumes of data with exceptional temporal and geographical granularity (Edwards, Housley, Williams, Sloan, & Williams, 2013), and our eagerness to harness this information has outpaced the adaptation of our traditional ethical frameworks (Williams, Burnap, & Sloan, 2017). With hindsight, researchers are starting to reflect on the ethical implications of this explosion of opportunity for research, for example, by examining users’ views of how they would expect their data to be used (Williams et al., 2018), providing frameworks for conducting ethical research (Townsend & Wallace, 2017), proposing processes for reproducing social media content in published work that protects private individuals (Williams et al., 2017), and tackling the thorny issue of sharing data for the purposes of replication without violating terms of service (Bishop & Gray, 2017).

Although this is a burgeoning area of research, the focus has been on the use of social media data *as an isolated source*. When social media data are linked with other forms of data, such as survey data, the issues are further complicated. For example, Twitter handles, the content of tweets and much of the metadata drawn down from the public application programming interface (API) allows an individual to be identified, but their “anonymization” would negate much of the additional insight offered. What then is to be done when Twitter data are linked with survey data where the data are not public and we would otherwise aim for anonymity?

In this article, we will explore such issues. Drawing upon our experiences of asking for consent to link survey and Twitter data on three studies (British Social Attitudes 2015; Curtice, Phillips, & Clery, 2016), the Understanding Society Innovation Panel 2017 (University of Essex, Institute for Social and Economic Research, 2018), and the NatCen Panel (Jessop, 2018), we address the following research question:

**Research Question 1:** What are the operational and practical implications for conducting ethical research with linked Twitter and survey data?

For a researcher looking to link survey and Twitter data, establishing informed consent is the most visible challenge (see Al Baghal et al., 2019), but the reality is that, even after informed consent has been given, there are a myriad of issues to be resolved concerning collection of the social media data, the environment in which the linkage can take place, what is and is not disclosive, and what can be archived privately and publicly for posterity, and how. Thus, the scope of this article is to explore what “good” ethical practice may look like in this context. In doing so, we purposefully avoid rehearsing debates related to the moral nature of ethics, and instead, we refer readers to other sources that outline how deontological, teleological, and virtue-based ethics apply to research using social media data (see Webb et al., 2017). This is not to say that these issue are not important, but rather that, in the light of ongoing public concern regarding the harm caused by the misuse of social media data, our focus is on how practical protocols and processes can minimize this.

## Context

Surveys have suffered from declining response rates, both in cross-sectional and longitudinal studies (Groves et al., 2009). At the same time, there has been a proliferation of data collected on individuals with information not frequently available in surveys. In response, efforts have been made to link these new sources of data to survey data to both address nonresponse and to add to the available data for analysis (e.g., Al Baghal, 2016; Eisnecker & Kroh, 2017; Mostafa & Wiggins, 2018; Sakshaug, Hülle, Schmucker, & Liebig, 2017; Sakshaug, Couper, Ofstedal, & Weir, 2012). To link these data, survey researchers have to ask permission from respondents, both to obtain information to locate records (e.g., Twitter handles) and for ethical reasons.

However, consent to these requests is far from universal, and there is high variation across studies. Reported consent rates have ranged from 19.0% (McCarthy, Shatin, Drinkard, Kleinman, & Gardner, 1999) to 96.5% (Rhoades & Fung, 2004). The choice to consent or not and the variation in rates is not fully understood, although studies have identified several factors. For example, Al Baghal (2016) reports that the same respondents provided lower consent rates for health records than for education records. It may be that some records, such as for health, are seen as more private than others, and privacy concerns are linked to lower consent rates (Sakshaug et al., 2012).

Respondents who are more risk averse or display lower trust levels, either indirectly (e.g., refusing to answer income questions) or directly (e.g., reports of trust in people), are also less likely to consent (Al Baghal, 2016; Sakshaug et al., 2012; Sala, Burton, & Knies, 2012). Other respondent characteristics are frequently (but not always) found to be related to consent. Several studies have found females less likely to consent (Knies, Burton, & Sala, 2012; Sala et al., 2012), as well as there being a number of findings that minorities are less likely to consent to linkage (Al Baghal, 2016; Kho, Duffett, Willison, & Brouwers, 2009; Knies & Burton, 2014; Knies et al., 2012; Mostafa & Wiggins, 2018).

Work on linking surveys to Twitter data is limited and in its nascent stages. The only published comparative study found that, compared with many other linkage requests, there was a relatively low consent rate for linking Twitter and survey data (Al Baghal et al., 2019). Examining consent rates across three U.K. surveys found that between 27% and 37% of respondents with personal Twitter accounts agreed to linkage. In two of these surveys, younger respondents consented at a lower rate than older, with women less likely to consent in one study with no difference found in the other two. Importantly, consent rates varied by survey mode, with respondents being asked to consent directly by an interviewer having higher consent rates than among respondents answering via the web.

## Informed Consent for Twitter Data Linkage

Ensuring that participants have agreed to take part in a study, and that this agreement was given with as full an understanding of what participation involves as possible, has long been fundamental to social research. Indeed, more recently, General Data Protection Regulation (GDPR) legislation may mean that consent is a legal requirement depending on a study’s legal basis for processing data (Information Commissioner’s Office, 2019a). For the purposes of this article, we are considering consent as (part of) an ethical basis for research, rather than a legal one (it is also the case that these studies were conducted before GDPR came into effect). If we consider an ethical approach to research as simply one that aims to maximize benefits but also minimize the risk of harm, then this is not necessarily at odds with a legal definition. However, in practice, consent as part of an ethical approach to research means we do not approach it as a fixed requirement or taking a fixed form, but as part of the research whole and alongside other ethical considerations—what is practical, the value and integrity of the research, and the risk of harm to participants. In contrast, using consent as a legal basis for processing data under GDPR begins to give it more fixed requirements (Information Commissioner’s Office, 2019b).

Although informed consent has historically formed an integral part of ethical social research, the use of big data in general (and social media data more specifically) has disrupted this (Williams et al., 2018). As the data have already been “collected” by the platform, there is no natural direct contact between the researcher and data subject at which informed consent for participation may be sought. For studies using social media data on a large scale, which may involve many of thousands or even millions of accounts, the feasibility of contacting those account holders to obtain consent is low. Many researchers will therefore point to the Terms of Service of platform providers as evidence of consent, but this is problematic for several reasons, in particular the low rates of users actually having read them (Beninger, Fry, & Jago, 2014).

Among the methodological advantages of linking social media data to survey data, this approach offers a way around these issues—there is “direct” contact between the researcher and the participant via the survey, affording the opportunity to communicate the study, and collect informed consent. However, this creates its own challenge of how to not only maximize consent rates (to maximize the representativeness of the sample) but also maximize the extent to which that consent is informed.

In the survey context, consent for data linkage is asked as part of a series of questions, and the framing of the questions is of particular importance, as is the communication channel (aural or visual) of the request. Research has shown that survey respondents tend to have limited understanding of all aspects of the consent request (Bates, 2005; Thornby, Calderwood, Kotecha, Beninger, & Gaia, 2018), and this may be particularly true in web surveys (Das & Couper, 2014; Sakshaug et al., 2017). Given the identified limitations, consent requests should be worded as simply as possible while still conveying enough information for informed consent, particularly when using a web (or other self-administered) mode.

In the medical context, Singleton and Wadsworth (2006) agree that some simplicity should be employed, and outline the types of information that need to be provided in a consent request. Information to be provided applicable to a social survey include description of research aims in lay terms, what information is needed from the respondent, that they can decline or change their mind, who can be expected to use the data, assurances of confidentiality and how this confidentiality will be maintained, and the timeframe where data will be collected from.

Of the three studies on which we asked participants for consent to link their survey and Twitter data (British Social Attitudes 2015; the Understanding Society Innovation Panel 2017 [IP10], and the NatCen Panel), IP10 and NatCen Panel used a sequential mixed-mode (web-first) survey. Participants were first asked if they had a Twitter account or not. Those that did were then asked for consent to link their Twitter data to survey responses, and those that consented were asked to provide their Twitter handle. The consent questions developed for these two studies were similar, reflecting the types of information laid out in Singleton and Wadsworth (2006). Acknowledging the possible difficulties in understanding, questions were asked in a straightforward way, leveraging the web design where possible to minimize misunderstanding.

The text of the consent request (included below) was worded with three key objectives in mind. First, it aimed to cover the types of information needed for informed consent (Singleton & Wadsworth, 2006)—(1) why we were collecting the data, (2) what we planned to do with the data, (3) what information we would collect, (4) that the data would be held securely, and (5) that they will not be identifiable in published information:

(1) As social media plays an increasing role in society, we would like to know who uses Twitter, and how people use it. (2) We are also interested in being able to add people’s, and specifically your, (3) answers to this survey to publicly available information from your Twitter account such as your profile information, tweets in the past and in future, and information about how you use your account.

(4) Your Twitter information will be treated as confidential and given the same protections as your interview data. (5) Your Twitter username, and any information that would allow you to be identified, will not be published without your explicit permission.

The second objective was to keep the wording broad. These questions were designed to collect consent to produce data sets that may be archived and of use to future research, for which details are not yet known. As such, the language used aimed to be as informative as possible, without being so specific as to limit the utility of the data. For example, we refer to information “*such as* your profile information . . .” In contrast, the specificity of “answers to *this survey*” is potentially limiting—both IP10 and the NatCen Panel are longitudinal studies, and this wording may be interpreted as insufficient consent to link to earlier or subsequent survey data collection waves.

The third objective was to keep the information presented relatively simple, to minimize the misunderstandings previously found (e.g., Bates, 2005; Thornby et al., 2018). In surveys, complex wordings and jargon are cognitively burdensome and less likely to be comprehended or even read (Tourangeau, Rips, & Rasinski, 2000). When linking to Twitter in particular, it is a challenge to communicate the detail of any consequent analysis because of the complexity of the methods that might be used (e.g., natural language processing, machine learning, etc.). We were concerned that were the information to become more complex, or even just longer, participants would be less willing to read, pay attention to, or understand it, making any consent *less* informed. As such, the information presented up-front was kept as simple as possible while allowing us to cover the key issues identified above. To leverage the web design, and try to further reduce misunderstanding found in this mode (e.g., Das & Couper, 2014) by ensuring the opportunity to find more detailed information, a series of hyperlinks were provided which allowed the participant or interviewer to access additional, more detailed, information (full text included for NatCen Panel in the appendix):

What information will you collect from my Twitter account?

What will the information be used for?

Who will be able to access the information?

What will you do to keep my information safe?

What if I change my mind?

## In What Way Is Twitter Data Disclosive?

The challenge then is to ensure that the conditions under which informed consent is granted are honored, but ensuring this requires an understanding of the technical and operational factors surrounding the collection, provision, parsing, and storage of Twitter data. In light of this, what follows is a description of the sheer amount of data available to researchers, how the data are accessed, and a detailed breakdown of a tweet record with a description of what the measure is and risk assessment of its disclosure potential. Alongside this is a discussion of how anonymity could be compromised through outliers (extreme values), correspondence between multiple measures, or as a result of the exact temporal granularity of Twitter data.

The simplest way to collect Twitter data is by using a tool, such as COSMOS (see http://socialdatalab.net/COSMOS), which has been designed to allow nontechnical users access to this rich and voluminous data source. Yet, the simplicity of such tools belies the complexity of the data that is being collected and the sheer amount of information that is associated with a single tweet, which can come with over 150 associated “attributes” (Twitter, 2019a) or, in the language of the social sciences, “variables.” As an example, if we collected 1,000 tweets from a user, we could have as many as 150,000 pieces of information relating to that individual and the content produced, from the color of their profile background to the language of their user interface. Although the latter two examples might not on their own present a disclosure risk, there are a plethora of other attributes that are unique to the tweet and the user (e.g., user ID or handle), place the user in a low-level spatial geography (e.g., geotagging with latitude and longitude), or might identify them as an outlier (e.g., an abnormally high number of followers). To fully understand how this occurs, it is necessary to take a shallow dip into the complexities of Twitter data.

Typically, Twitter data are accessed through an API and encoded using JavaScript Object Notation (JSON). The API can be used to pull down information on objects such as *Tweets* and *Users* and will include a wide array of information, some unique to the object itself and others more generic. For example, a *Tweet* will always have a unique ID, an author, the content of the message itself, and details of when it was posted. Some *Tweets* are geotagged and include information regarding the latitude and longitude of where the tweet was published, and others contain hashtags, images, and mentions. The key point is that when we pull down a tweet we also receive a deluge of additional information, and this happens *with every single tweet*. For example, if we pulled down two tweets from the same user, we would receive all the additional information both times, even if the values in the other attributes have not changed (assuming they are not null). From a computational processing point of view, this is convenient, reduces the need for relational databases, and speeds up data processing—from a memory point of view it means that Twitter data collections are often much larger than a researcher anticipates. Either way, the constant rerecording of the same attributes does open up new avenues for research as we can see how perhaps initially unimportant things change over time, such as how users describe themselves or how their follower and followee numbers change in light of their behavior. Indeed, this comprehensive repeated snapshot is precisely why Twitter data can add value to survey studies through data linkage, but it also presents a substantial problem in that many researchers may not be aware of the extent of information they are collecting about an individual and what attributes could compromise respondent anonymity.

Table 1 provides a summary of selected attributes associated with Twitter JSON, whether they relate to tweet or user or geographical information, what they are, the nature of the risk, and an overall assessment of the risk of individual identification. A full table with all possible attributes would be over 150 rows in length, so here we have presented the 43 attributes (at the time of writing) generated by the “streamR” package for R (Barbera, 2018). These attributes are typically the *most relevant attributes* that may be of use to a researcher in the social sciences. The Twitter data dictionary (Twitter, 2019a) was used to help define attributes and the definitions have been adapted for readers without a technical background where appropriate. Not all attributes are labeled the same on both streamR and the Twitter data dictionary—where a difference occurs we have defaulted to the streamR label. The disclosure risk of each attribute has been assessed in isolation and where the outcome is not simple or clear-cut, we have used the label “VARIABLE.” Where an attribute is, for the most part, likely to be nondisclosive except in rare (but realistic) cases, we have identified this caveat with an “*.”

**Table 1.** Analysis of Disclosure Risk by Twitter JSON Attributes.

Relating to	Attribute	Description	Nature of risk	Risk of identifying an individual
Tweet	text	The actual text of the tweet	If not a retweet, then unique content and directly identifiable	High
Tweet	retweet_count	The number of times a tweet has been retweeted	Changeable and dynamic, unlikely to be unique *unless extreme	Low*
Tweet	favorite_count	The approximate number of times a tweet has been liked by other users	Changeable and dynamic, unlikely to be unique *unless extreme	Low*
Tweet	favorited	Indicates whether a user has favorited the tweet	Binary categorical variable, common practice to “favorite” a tweet	Negligible
Tweet	truncated	Whether a tweet text has been truncated (greater than 140 characters)	Binary categorical variable, truncation common with new 280 character tweet limit	Negligible
Tweet	id_str	The numeric (string) version of the unique identifier for this tweet	Unique content, directly identifiable—often deposited to allow other researchers to “rehydrate” Twitter data sets	High
Tweet	in_reply_to_screen_name	If the tweet is a reply to another tweet, this is the name of the original tweet’s author	Evidence of Twitter correspondence with another unique user, may or may not represent someone in their network, often used for responding to public individuals (e.g., politicians) but could also be used to respond to users who are closely connected	Variable
Tweet	source	The utility used to post the tweet (e.g., Tweets posted from the Twitter website have a source of “web”)	Unlikely to pose a risk as alternative Twitter posting tools are in widespread use	Negligible
Tweet	retweeted	Indicates whether the tweet has been retweeted by the user	Binary categorical variable, common practice to retweet	Negligible
Tweet	created_at	Creation date and time of the tweet to the second (in UTC) (e.g., Tuesday November 23 12:46:54 +0000 2018)	On average there are 6,000 tweets created every second (http://www.internetlivestats.com/twitter-statistics/), and it is difficult (if at all possible) to acquire all historic tweets made in a given second without access to the firehose (100% feed). Note that offset (“+0000”) could be used to determine time zone (but see later comment on GDPR)	Low
Tweet	in_reply_to_status_id_str	If the tweet is a reply to another tweet, this is the ID of the original tweet	Represents part of a conversation that the user is partaking in could be used to identify an individual if number of responses to original tweet are small	Variable
Tweet	in_reply_to_user_id_str	If the tweet is a reply to another tweet, this is the ID of the original tweet’s author	Evidence of Twitter correspondence with another unique user may or may not represent someone in their network, often used for responding to public individuals (e.g., politicians) but could also be used to respond to users who are closely connected	Variable
Tweet	lang	The language of the tweet text (machine-detected)	Machine detection will allocate to one language or mark as “undetected,” will only identify a single language, might well not be the same as language of interface, can change with every tweet (dynamic) *but might result in “low cell count problem” for minority languages	Negligible*
Tweet	expanded_url	Full (expanded) version of a URL included in the tweet	Depends where the URL points to, often to generic content (e.g., BBC News story) but could be to personal website or blog	Variable
Tweet	url	Wrapped URL corresponding to the value directly embedded into the raw tweet text	Depends where the URL points to, often to generic content (e.g., BBC News story) but could be to personal website or blog	Variable
User	listed_count	The number of public lists that the user is a member of	Unlikely to be unique *unless extreme	Low*
User	verified	Whether account has been verified (account of “public interest”)	Binary categorical variable, not unusual and could include actors, musicians, journalists, politicians, organizations, and so on.	Negligible
User	location	The location defined by the user	May or may not represent where the user lives or works, but potentially could place user in a low-level spatial unit	Variable
User	user_id_str	The numeric (string) version of the unique identifier for this user	Unique identifier, directly identifies the user	High
User	description	User-defined description of their account, often used as a “bio”	Regardless of what the user writes, this is likely to unique to the individual	High
User	geo_enabled	User has enabled the possibility of geotagging their tweets	Simply enables geotagging, does not enforce it. Binary categorical variable—research suggests that 41.6% of users have this setting enabled (Sloan & Morgan, 2015)	Negligible
User	user_created_at	Creation date and time of the user account to the second (in UTC) (e.g., Tuesday November 23 12:46:54 +0000 2018)	Potentially unique to the individual due to high level of temporal granularity, note that offset (“+0000”) can be used to determine time zone (but see later comment on GDPR)	High
User	statuses_count	The number of tweets and retweets posted by the user	Changeable and dynamic, unlikely to be unique *unless extreme	Low*
User	followers_count	The number of followers the user account currently has	Changeable and dynamic, unlikely to be unique *unless extreme	Low*
User	favourites_count	The number of tweets the user has favorited since the account was created	Changeable and dynamic, unlikely to be unique *unless extreme	Low*
User	protected	Whether account is protected (tweets only visible to followers)	Binary categorical variable, not unusual practice	Negligible
User	user_url	A URL given by the user, normally a link to a personal/organizational website	Not necessarily unique, but will be in some cases, not unusual for users to direct to personal websites	High
User	name	The self-defined name of the user	Not necessarily the name of a person, but often is	High
User	time_zone	The time zone of the user	If present will place the user in a large-scale geography, but from May 23 has been returned as “null” (private field) due to EU privacy laws	NA
User	user_lang	The user’s choice of interface language	Twitter is available in 47 languages (at time of writing), may well not be the same as the language in which tweets are written, can change but most likely to be static	Negligible
User	utc_offset	The difference in hours and minutes between user time zone and UTC	If present will place the user in a large-scale geography, but from May 23 has been returned as “null” (private field) due to EU privacy laws	NA
User	friends_count	The number of accounts this user is following	Changeable and dynamic, unlikely to be unique *unless extreme	Negligible*
User	screen_name	The screen name (aka handle) of a user	Screen name can change (dynamic) but is always unique, an individual identifier	High
Geo	country_code	Two letter code of the country a tweet was issued from, or is about	May be derived from an exact point coordinate (lat/long), or from a place selected by a user such as a city. In the latter, this may be the country of the place from where the user is tweeting from, or a place that they are tweeting about. Either way, on its own this represents a high-level geography	Negligible
Geo	country	Name of the country a tweet was issued from or is about	May be derived from an exact point coordinate (lat/long), or form a place selected by a user such as a city. In the latter, this may be the country of the place from where the user is tweeting from, or a place that they are tweeting about. Either way, on its own this represents a high-level geography	Negligible
Geo	place_type	The nature of the location the tweet was issued in, or is about, such as a city or POI	Classification of place identified by user (either selected or derived from point coordinates) is generic and unlikely to be problematic	Negligible
Geo	full_name	Full name (string) of place, for example, “San Francisco, CA”	Could lead to low-level spatial data if point coordinates, or user selection, results in identifying a city or town	Variable
Geo	place_name	Short name (string) of place, for example, “San Francisco”	Could lead to low-level spatial data if point coordinates, or user selection, results in identifying a city or town	Variable
Geo	place_id	Unique ID (string) of place	Could lead to low-level spatial data if point coordinates, or user selection, results in identifying a city or town	Variable
Geo	place_lat	Center point of the location the tweet was issued in, or is about (latitude)	Gives a latitude value at the centroid of the location (e.g., center of Manchester), may or may not be where the user was when tweet was posted, unlikely to be of use without corresponding longitude value	Low
Geo	place_lon	Center point of the location the tweet was issued in, or is about (longitude)	Gives a longitude value at the centroid of the location (e.g., center of Manchester), may or may not be where the user was when tweet was posted, unlikely to be of use without corresponding latitude value	Low
Geo	Lat	Latitude of tweet location	Precise latitude of where user was when they tweeted, potentially could be at home or work, alternatively may be commuting. Either way has considerable potential to locate individuals in low-level geographies, but this is significantly reduced without longitude value *risk is considerably higher with corresponding longitude	Medium*
Geo	Lon	Longitude of tweet location	Precise longitude of where user was when they tweeted, potentially could be at home or work, alternatively may be commuting. Either way has considerable potential to locate individuals in low-level geographies, but this is significantly reduced without latitude value *risk is considerably higher with corresponding latitude	Medium*

*Note.* JSON = JavaScript Object Notation; GDPR = General Data Protection Regulation; POI = point of interest; EU = European Union.

Alongside the obvious candidates for identifying an individual (individual tweets, tweet IDs, screen name, and user IDs), there are several attributes that are problematic. The exact time and date that a user account was created is set in stone and not dynamic, so it could be used to identify a single user or a small group. Descriptions of profiles (biographies) are dynamic but nevertheless are likely unique and could be matched with historic records of tweets to identify an individual, in addition to the fact that the substantive content itself might include an identifier (e.g., “I’m a researcher at University X specializing in Y”). User URLs might refer to organizational webpages that may or may not identify an individual, and responses to other tweets and mentions may correspond to colleagues, family, and friends. Even metrics such as number of followers, followees, tweets, and lists may be disclosive if the values are extreme enough, which is precisely why public survey data typically does not report variables such as income at the interval level.

An additional complication is that although the attributes have been discussed in isolation here, the reality is that a researcher accessing this data will have access to more than one attribute value at a time. As with a survey, only the most granular variables might prove disclosive on their own (such as annual pay to the nearest £), but often the risk comes from obtaining low cell counts through the crosstabulation of several variables at once. In the case of Twitter data, knowing the longitude of a tweet offers a geographical range, but adding latitude gives a point coordinate. Less obvious problems may arise from metric data that is well within the normal range. For example, knowing that someone has 456 followers is unlikely to make them identifiable, but cross-reference this with knowing that they follow 578 people, are on 34 lists, and have favorited 132 posts and you may be down to a very small group of users who fit these criteria. Even the fact that these values are dynamic and change over time does not protect against disclosure risks because these data are returned within the JSON format every time someone tweets, and tweets come with an exact time and date of posting. In short, not only do we know the metric data, we also have a proxy time and date for when it was correct. Given access to the right data and enough time, it is feasible that someone could unpick all of these conditions and identify an individual if they were suitably motivated and technically skilled enough to do so.

Finally, it is important to note that these are only the fields listed (“parsed”) as part of the StreamR package. The data obtained through Twitter from which this summary has been extracted will still contain many more attributes, some of which are nested such as “extended_text” for tweets longer than 140 characters. This original data will still be on a machine, meaning that even after deleting problematic fields from the derived data above there will still be disclosive fields stored locally, sometimes as hidden files. It is therefore essential that proper secure protocols are put in place.

## Maintaining Security and Minimizing Risk

Alongside informed consent, the anonymization of data has been a core part of traditional social research methodologies. However, as with informed consent, the nature of social media data make it difficult to enforce these principles (Williams, Burnap, Sloan, Jessop, & Lepps, 2018). As outlined above, Twitter data, in their raw form, are inherently identifiable, even when obvious identifiers such as user IDs are removed, and anonymization may limit what analysis can be done. Although the use of raw Twitter data may be appropriate if it is considered “public data” (although the public/private nature of online environments is contested—see Joinson, 1998; Williams, 2006), the linking of raw Twitter data to survey data would also make the survey data identifiable. A particular ethical challenge of working with linked survey and Twitter data is how to maintain data security without reliance on anonymization.

The precise process for accessing data for analysis in a secure manner while maintaining security should be somewhat flexible to account for the varying nature of study requirements. Some research questions may not require access to identifying information for analysis to be conducted, and so the processes may differ somewhat. Table 2 below outlines four suggested areas where data security should be considered when processing linked survey and Twitter data for analysis: systematic processing, data reduction, controlled access, and data deletion.

**Table 2.** Principles for Maintaining Security (Linked Twitter and Survey Data).

1. Systematic processing	As much as possible, data should be managed in a systematic and considered manner. Based on the processes used for linking survey and administrative records (Administrative Data Research Network, 2018), once initial consent has been collected, survey data and Twitter data should be stored and processed separately until data linkage is required, to help control access and minimize the risk of disclosure.
2. Data reduction	To conduct analysis for any given research question, it is likely that not all of the available survey and Twitter data need to be linked together. As such, only the survey and Twitter data necessary for analysis should be made available for linkage. For the survey data, by only linking the answers required, we reduce the amount of information that may be linked back to an individual person, and therefore the risk of harm. For the Twitter data, reducing the linked variables may reduce the ease with which someone with access to the data might be able to identify a person. Should the “high-risk” variables be excluded from the linked analysis then the risk may be reduced substantially. As well as reducing the number of variables linked, data reduction may take the form of the creation of derived variables. For example, while the analysis may require raw Tweet content initially, the linked analysis may only require a derived variable indicating whether or not a Tweet contained a reference to a particular topic, which is less likely to be individually identifiable.
3. Controlled access	Throughout the data management process, access to identifiable data should be limited to those who need it to minimize the risks of disclosure. The linked data should be held securely, so that access is granted only to those who need it, and those people with access should be documented and have appropriate training for working with identifiable data.
4. Data deletion	Data should only be held for as long as is necessary for analysis to be conducted. Once the project is complete, as with other forms of personal data, data should be securely deleted and archived if necessary.

**Figure 1.**
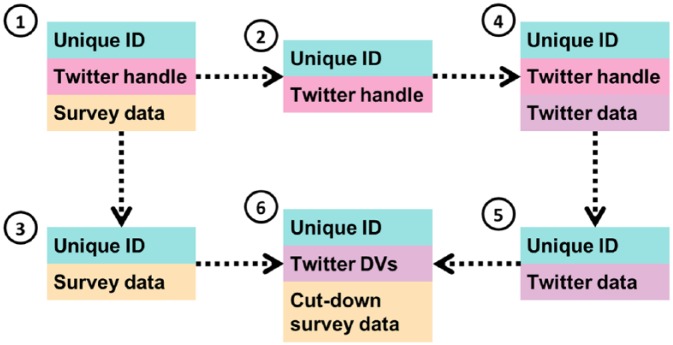
Data flow diagram for linking survey and Twitter data.

Thinking more practically, Figure 1 outlines one way of processing data securely, and reflects the process used for linking NatCen Panel members’ data to their Twitter data in a project to understand political behavior (Jessop, 2017). It is based on processes developed for securely linking survey and administrative data (Administrative Data Research Network, 2018), reflecting the principle of “systematic processing.” Initially, the data collected from the survey will include a unique ID, the survey data, and the panel member’s Twitter handle (if they have consented and provided it) (1). The initial data processing should split this data into two—separating the (identifying) Twitter handle from the survey data into two data sets—(2) and (3), respectively—with both carrying a unique ID which would allow them to be matched back together.

At this point, the data can begin to be analyzed. For the Twitter data, this will initially involve using the collected Twitter handles to request the panel members’ data from the Twitter API (4). Although this is still the “public” Twitter, caution should still be used here—the data are identifiable and all members of the sample are being identified as survey participants—information that they would not have made public. Consideration still should therefore be made as to where these data are stored and who has access at this point, and to begin the process of data reduction to minimize risk, for example, dropping Twitter handles (5), but also perhaps Twitter API data that are not required for this analysis.

Once the Twitter data have been downloaded, and the handles dropped, the two data sets may be linked back together for analysis (6). This should happen in a secure environment, reflecting the principle of “controlled access.” In this instance, both data sets were made available to the researcher in the secure data enclave at NatCen’s offices in London, which is used to analyze personally identifiable information, and has strict procedures such as controlled and documented access, and restrictions regarding what can be taken into and out of the lab.

However, under the principle of “data reduction,” it is preferable that it is not the full survey and Twitter data sets that are linked together for analysis. For example, for this study, although more detailed analysis was conducted on the survey data independently, only age, how people voted, and a classification based on latent class analysis were required for the linked analysis. In addition, precise age was not required, and some parties were not voted for in sufficient quantities to be useful for analysis, so these categories could be collapsed. By going through this process, we reduce both the risk of disclosure, but also the risk of harm should disclosure occur, as less information would be able to be linked to an individual.

Similarly, the Twitter data may also be reduced to diminish the risks of disclosure and harm. This may involve the dropping of nonessential individual variables collected via the Twitter API, but it could also be through the use of derived variables rather than the raw data. For example, for this study, raw Tweet content was needed to classify a Tweet as “pro-Labor” or “anti-Labor,” but for the linked analysis, only the classification of the tweet was required rather than the full text, so only that was made available for linkage, substantially reducing the risk of disclosure and harm.

Finally, for this project, once the analysis had been completed, the data were deleted. As the data were processed in a systematic manner, the locations of potentially disclosive data sets was known, and we were able to delete them. At the same time, data sets (2) and (3) from Figure 1, which are not inherently disclosive, have been kept and stored securely so that the analysis can be reproduced should that be required or further work be considered.

Additional specific systems can be used to ensure data security and minimize risk. For example, the IP and its related data are protected using an information security management system (ISMS). These systems are designed to protect information along three dimensions: confidentiality (protection against unauthorized disclosure and use), integrity (protection against unauthorized modification), and availability (protection against unauthorized destruction). These types of systems can be, and the system housing the IP has been, certified by the International Organization for Standardization (ISO). The ISMS housing IP and its related data have the ISO27001 certification. ISO27001 “specifies the requirements for establishing, implementing, maintaining and continually improving an information security management system within the context of the organization” (ISO, 2013).

## Archiving and Reuse

The archiving and sharing of research data are important elements of the research process and often a requirement for funding organizations or research publications. It allows not only for the replication and verification of findings but also for the reuse of data to expand the research or explore entirely separate topics without the need to burden new or existing participants with additional data collection. Archiving social media data in isolation is not without its challenges (although see Kinder-Kurlanda, Weller, Zenk-Möltgen, Pfeffer, & Morstatter, 2017, for an excellent example of how to archive geotagged Twitter data), so it follows that archiving linked survey and Twitter data is even more complex.

The processes for archiving linked social media and survey data and making it available for reuse should, in principle, follow the same framework outlined above, and again build on established processes for secure data linkage. Consent questions should be worded to ensure participants are aware that data may be archived. Furthermore, security should be maintained and risk of harm minimized through controlling access and data reduction, and following secure data management and deletion protocols appropriate to the nature of the data being archived or accessed. However, there are potentially additional complications within this context.

The current terms of use for Twitter data prevent the sharing of data sets larger than 50,000 Tweets beyond the user (or their research team) who initially access the data. For studies that fall into this category, this would likely mean that raw Twitter data would not be able to be legally archived and shared. However, it is possible to share and archive tweet and user IDs. These can act as “dehydrated” forms of the data, which can be used by researchers to query the Twitter API and access the raw data, “rehydrating” it. Indeed, Twitter make special provisions regarding sharing tweet IDs for academics conducting noncommercial research (Twitter, 2019b).

One consequence of this approach is that should a user delete their account or a tweet that was part of any initial analysis, it will not be included in the “rehydrated” data set. In some regard, this is positive, as we might view such a deletion as a withdrawal of consent, and these cases should be excluded from the data set. However, for the purposes of replication, it means that researchers reaccessing the data may not be working with the same information that the original analysis was based on.

Enabling access to any data requires some level of work for those responsible for curating it, particularly where those data include identifiable information (e.g., removing data where consent is withdrawn, or setting up access in a secure environment and reviewing outputs taken out of a secure environment for disclosure risk). However, the nature of Twitter data and its analysis creates novel challenges. Depending on the context, the data analysis may require specific software, and many social media analysis tools are web-based. Even if this were not the case, the “rehydration” of Tweet IDs would require Internet access to query the Twitter API. This raises the question of whether an environment can be considered “secure” if the user accessing the data has access to the Internet. If not, how can that Internet access be controlled to minimize risks of abuse? Alternatively, is it possible for the data curator to rehydrate the data on the data user’s behalf and provide a local install of the required software, and is the amount of work required to do so appropriate?

Although the British Social Attitudes, Understanding Society Innovation Panel, and NatCen Panel studies referenced in this article have, to varying extents, been used for some form of linked analysis, none have gone through a formal archiving process and been accessed by researchers working independently of the original research team. Although we have identified what we think may be key issues and how they may be overcome, it will only be through actually archiving and providing access to these data that we might fully understand the challenges and whether or not the measures we have outlined will address them.

## Conclusion

Linked survey and Twitter data provide an unprecedented opportunity for social scientific analysis, but our ability to collect and collate data risks outpacing our technical understanding of how Twitter data are constituted and the ethical implications of its use. Although easy access to the data through the development of user-friendly tools has increased the use of Twitter data among social scientists, this has come at a cost. Simple graphical interfaces belie the complexity of the data and often give little clue as to the extent of what is actually being downloaded by only presenting a limited array of variables. This problem is compounded by misunderstandings by many researchers who are, entirely understandably, not aware of what is going on behind the scenes. In a very real sense, a little knowledge (knowing how to access the data) is a dangerous thing. This article has focused on the challenges specific to linked data projects and Table 3 provides a summary response to the research question (what are the operational and practical implications for conducting ethical research with linked Twitter and survey data?), but of course even a project focusing only on Twitter data might have good reason to follow the principles of anonymity and observe standard data security practices (Williams et al., 2017).

**Table 3.** Summary of Considerations for the Ethical Linkage of Survey and Twitter Data.

1. Consent	Large-scale social media data collection disrupts “traditional” approaches to collecting informed consent, as the increased distance between researcher and research participant, and the scale of the number of participants, can make that interaction impractical, while the “public” nature of the data and agreement to platform “Terms of Service” calls into question the necessity of further consent. However, in the context of linking survey and Twitter data, researchers have an opportunity to communicate directly with participants and to collect informed consent and should therefore do so. The researcher should aim for this consent to be as informed as possible, which will involve balancing being detailed with using simple language and not overwhelming the participant with information. For consent to be informed, information provided should cover: why the data are being collected; what will be done with the data; what data will be collected; how the data will be stored; what the risks of disclosure might be.
2. Disclosure	Due to its “searchable” nature, unlike traditional quantitative and or qualitative data, Twitter data often cannot be anonymized without a substantial loss in utility. As a result, anonymization should not, in most instances, be relied upon as a means of maintaining data security. This is potentially problematic for Twitter data collection in general, but is particularly challenging where it is linked to survey data which the participant has not chosen to put into the public domain. Understanding these risks necessitates better familiarization of what Twitter data are and what exactly an API is providing.
3. Security	Given the complexity of linking survey and Twitter data, the resulting difficulty in achieving truly “informed” consent, and the inability to consistently rely on anonymization of data to protect participants from risk of harm, increased emphasis should be placed on maintaining the security of data throughout the research process. By ensuring that the data management process is systematic and considered, controlling and limiting what data are made available, to whom, and in what environment, and securely deleting data when they are no longer required, data security can be enhanced and the risk of disclosure, or risk of harm should disclosure occur, reduced.
4. Archiving	The archiving or sharing of linked survey and Twitter data for further analysis carry the same problems as the initial processing of the data. As such, this may be done ethically should informed consent have been obtained and the data are archived or shared in a systematic and controlled manner. However, there are additional considerations for sharing Twitter data: Twitter’s Terms of Service prevent the sharing of large data sets beyond research teams. This can be circumvented by “dehydrating” the data to Tweet IDs, sharing these, and then “rehydrating” them to the full data. However, should those original Tweets or the accounts they came from have been deleted, they may not appear in the new data set, making the exact replication of the original data set impossible.

Note. API = application programming interface.

Even with full knowledge of the vast array of data provided from Twitter, changes in legal requirements, such as the recent introduction of GDPR, can alter what is and is not acceptable and even if the regulatory context is relatively stable, social media platforms can (and do) change the nature of the data provided through APIs, the Terms of Service for users, and the conditions listed in developer agreements.

Despite all of this, the fundamental principles of conducting ethical social research remain the same regardless of technological innovation and societal change. Indeed, the principles laid out by The Belmont Report (1979) still hold true: respect for persons (respecting autonomy through ensuring informed consent), beneficence (avoiding harm through avoiding disclosure and observing secure data practices), and justice (democratizing access through data archiving). In traditional modes of social research, these principles could only credibly be observed by researchers with an understanding of the nature of the data being collected, and the situation is no different for Twitter data. As a community of researchers, we have a clear duty to continue to explore and publicly discuss how we approach these problems in the spirit of promoting knowledge and good practice.

By reflecting and bringing to the fore the key issues associated with linked Twitter and survey studies, we hope to problematize the matter in a constructive manner and provide guidance on how future studies could proceed. The detailed breakdown of how Twitter data could be disclosive can be replicated for data from any social media platform, although special concern should be given to the specific problem of disclosure through multiple variable values. Moving forward, we encourage the academic community to consider the issues around secure access to linked data and how such a resource can be used more widely by researchers while not requiring unreasonable resources. Ultimately, through demonstrating the viability of linked studies, we hope that researchers can pose research questions that hitherto could not be addressed and proceed along new avenues of inquiry confident in the ethical integrity of their endeavor.

## Appendix

### Consent Questions for Linking Survey and Twitter Data, NatCen Panel, July 2017

#### TwitHas {ask all}

Do you have a personal Twitter account?

1. Yes
2. No

#### TwitConsent {IF TwitHas = yes}

As social media plays an increasing role in society, we would like to know who uses Twitter, and how people use it. We are also interested in being able to add people’s, and specifically, your answers to this survey to publicly available information from your Twitter account such as your profile information, tweets in the past and in future, and information about how you use your account.

Your Twitter information will be treated as confidential and given the same protections as your interview data. Your Twitter username, and any information that would allow you to be identified, will not be published without your explicit permission.

### HELP SCREEN: What Information Will you Collect From My Twitter Account?

We will only collect information from your Twitter account that is publicly available. This will include information from your account (such as your profile description, who you follow, and who follows you), the content of your tweets (including text, images, videos and web links), and background information about your tweets (such as when you tweeted, what type of device you tweeted from, and the location the tweet was sent from).

We will collect information from your past tweets (up to the last 3,000) and will update this with information from more recent tweets on a regular basis.

### HELP SCREEN: What Will the Information Be Used For?

The information will be used for social research purposes only. Adding your Twitter information and your survey answers will allow researchers from universities, charities, and government to better understand your experiences and opinions.

For example, using extra information from your Twitter account, researchers can start to

- Understand who uses Twitter and how they use it
- See what Twitter information can tell us about people, and how accurate it is
- Know what people in the United Kingdom are saying about things we don’t ask in our survey
- Look at additional information related to questions asked in the survey

### HELP SCREEN: Who Will be Able to Access the Information?

Matched data which includes both your survey answers and Twitter information will be made available for social research purposes only. Researchers who want to use your matched Twitter and survey information must apply to access it and present a strong scientific case to ensure that the information is used responsibly and safely.

Matched statistical information from your Twitter account which you cannot be identified from (e.g., how often you Tweet, or whether you follow any politicians) will have the same access controls as your other survey answers.

At no point will any information that would allow you to be identified be made available to the public.

### HELP SCREEN: What Will You Do to Keep My Information Safe?

All information we collect will be held in accordance with the Data Protection Act 1998.

Because Twitter information is public data that anyone can search, it is impossible to anonymize completely. To keep your information safe, researchers will only be able to access the matched survey answers and detailed Twitter information in a secure environment set up to protect this type of data. Only approved researchers who have gone through special training may access this information, and they will have to apply to do so.

Statistical information from your Twitter account which you cannot be identified from (e.g., how often you Tweet, or whether you follow any politicians) will have the same level of protection as your other survey answers.

### HELP SCREEN: What if I Change My Mind?

This information will be collected and stored for as long as they are useful for research purposes, or until you contact us to withdraw your permission. You can do this at any time by emailing us at panel@natcen.ac.uk or calling 0800 652 4569, and do not have to give a reason.

{END OF HELP SCREENS}

Are you willing to tell me your personal Twitter username and for your Twitter information to be added to your answers to this survey?

1. Yes
2. No

#### TwitUsername {IF TwitConsent = Yes}

What is your Twitter username?

[OPEN]

SOFTCHECK: “Twitter usernames must begin with an @ character, followed a maximum of 15 characters (A-Z, a-z, 0-9, underscore), no word spaces. Please check and amend.”
